# Paternal Glufosinate Ammonium Exposure Leads to Memory Dysfunction in Offspring Mice

**DOI:** 10.3390/toxics14050396

**Published:** 2026-05-06

**Authors:** Zhilu Pei, Dayu Hu, Jie Sun, Weiyue Hu

**Affiliations:** 1 Key Laboratory of Modern Toxicology of Ministry of Education, School of Public Health, Nanjing Medical University, No.101 Longmian Road, Nanjing 211166, China; peizhilu@stu.njmu.edu.cn (Z.P.); hudayuu@163.com (D.H.); 2 Department of Nutrition and Food Safety, School of Public Health, Nanjing Medical University, Nanjing 211166, China; 3 Department of Endocrinology, Endocrine and Metabolic Disease Medical Center, Nanjing Drum Tower Hospital, Affiliated Hospital of Medical School, No.22 Hankou Road, Nanjing 210093, China; 4 Branch of National Clinical Research Centre for Metabolic Diseases, Nanjing 210093, China

**Keywords:** glufosinate ammonium, developmental toxicity, neurobehavior, microglia, neuroimmune

## Abstract

Background: Glufosinate ammonium (GLA) is a widely used herbicide, yet potential neurodevelopmental risks related to paternal exposure before conception remain insufficiently defined. Methods: In this study, adult male C57BL/6J mice received GLA at 0.2 mg/kg·day for 10 consecutive weeks and were then mated with unexposed females to generate F1 offspring. Offspring growth was monitored, and neurobehavior was assessed at 5 weeks of age. Results: In behavioral tests, female offspring showed reduced social novelty preference in the three-chamber test and impaired spatial learning and memory in the Morris water maze test, while open field, elevated plus maze, and rotarod performance were not altered. Male offspring showed no clear group differences in these memory-related endpoints. Golgi staining revealed reduced dendritic complexity and spine density in the hippocampus and prefrontal cortex. Glial markers were elevated, and neuronal marker changes showed region-dependent shifts. TUNEL staining indicated increased apoptosis during embryonic development and persistent apoptotic signals in the juvenile prefrontal cortex, accompanied by cytokine imbalance with increased IL-1β and decreased IL-10 in the hippocampus. Conclusion: These results suggest that paternal preconception GLA exposure is associated with selective memory-related behavioral deficits in juvenile offspring and with convergent glial, inflammatory, and apoptosis-related brain changes. These findings support the consideration of paternal exposure in developmental risk assessment frameworks.

## 1. Introduction

Glufosinate ammonium (GLA) is an organophosphate herbicide widely used in agriculture and in public domains. Its use is often discussed alongside glyphosate because herbicide tolerant genetically modified crops have been engineered to resist these agents. The extensive use of GLA has raised concerns about population exposure, which is supported by biomonitoring studies that have quantified GLA in human matrices, including urine and plasma [1]. In addition, a pregnancy cohort study that measured urinary concentrations of glyphosate, GLA, and their primary metabolites highlights that biomonitoring data for these herbicides are sparse and motivates further toxicological evaluation of potential developmental hazards [2].

Against this exposure backdrop, experimental studies have provided evidence that developmental exposure to GLA can produce persistent neurobehavioral outcomes. In our previous study of prenatal GLA exposure, offspring displayed autism-like behavioral features, supporting neurodevelopmental vulnerability under early life exposure conditions [3]. Mechanistic studies indicate that GLA can engage targets relevant to brain function. GLA inhibits mammalian glutamine synthetase in vitro and ex vivo, and in the central nervous system this enzyme is reported to be exclusively localized in glial cells, suggesting that glial biology may be sensitive to GLA [4]. Acute neurotoxic effects are supported by a seizure model in which GLA-induced repetitive tonic–clonic seizures and epileptic discharges appeared simultaneously in the hippocampus and cerebral cortex [5]. Long-term effects during chronic exposure at low doses have also been considered as high field MRI revealed structural changes in the hippocampus and somatosensorial cortex after repeated dosing over ten weeks [4].

Developmental windows appear particularly sensitive to GLA as pre- and postnatal exposure to low dose GLA was reported to influence early reflex development and affiliative behaviors in mice [6]. Perinatal exposure has been shown to impair neurogenesis and neuroblast migration [7]. In vitro work using murine primary neural stem cell cultures further showed that GLA and its major metabolite altered neuro-glial differentiation and disturbed ependymal wall integrity in the ventricular subventricular zone [8]. Mouse embryos in culture also demonstrated GLA-induced apoptosis in the early neural tissue of neuroepithelium [9]. Inflammation is a candidate pathway since GLA exposure increased interleukin 1 beta (IL-1β) in an IL-1R1-dependent manner [10], and prior inflammatory sensitization increased vulnerability to low dose GLA exposure in a multiple-hit design [11].

Although the developmental literature is growing, most GLA studies emphasize maternal or direct offspring exposure, and considerably less is known about paternal exposure before conception. A review of paternal preconception exposures highlights that paternal environmental factors can influence offspring health outcomes independently of direct fetal contact [12]. Rodent studies provide examples that paternal exposures can alter cognition- and memory-related behaviors after paternal glucocorticoid exposure prior to mating [13] and modified fear memory after chronic paternal nicotine exposure [14].

Collectively, available evidence supports neurodevelopmental sensitivity to GLA under direct developmental exposure conditions, yet the consequences of paternal GLA exposure remain insufficiently defined. In the present study, we built a paternal preconception GLA exposure model to test whether paternal GLA exposure contributes to neurobehavioral vulnerability of F1 offspring generated by natural mating. Because currently available human biomonitoring data for GLA and its major metabolite remain limited, especially for occupational and male internal exposure, the paternal exposure level used here was selected as a biologically interpretable dose with reference to the ADI, which was also chosen in continuity with our previous studies on paternal GLA exposure. This focus on paternal preconception exposure distinguishes the present study from the majority of previous GLA studies, which mainly emphasize maternal, perinatal, or direct developmental exposure paradigms. We focused on neurobehavior at five weeks of age and prioritized memory-related domains, given evidence for epigenetic alterations in GLA exposure paradigms [15,16]. By providing phenotype-level evidence that links paternal GLA exposure to direct offspring neurobehavioral and neuropathological changes, this work helps clarify paternal contributions to neurodevelopmental toxicity of a widely used herbicide and supports the consideration of paternal exposure in developmental risk assessment frameworks.

## 2. Materials and Methods

### 2.1. Animals and Ethics

C57BL/6J mice were provided by the Experimental Animal Center of Nanjing Medical University. Animals were housed in a SPF barrier facility (24 ± 1 °C, 45 ± 10% relative humidity, 12 h light/dark cycle) with ad libitum access to standard chow and water and wood for gnawing to relieve anxiety. All procedures were approved by the Institutional Animal Care and Use Committee of Nanjing Medical University (approval no. IACUC-2201008) and were performed in accordance with the ARRIVE 2.0 guidelines.

### 2.2. Chemical and Exposure Preparation

Glufosinate ammonium (GLA; CAS 77182-82-2; analytical standard) was purchased from Aladdin (Shanghai, China). For paternal exposure, the GLA group received GLA at 0.2 mg/kg/day via drinking ultrapure water. The final working concentration was calculated from the daily water consumption [3] and the average body weight of mice within each cage. The selected dose was aligned with the low dose used by Laugeray et al. [7]. After body surface area adjustment [17], this regimen corresponds to an estimated human equivalent dose of approximately 0.016 mg/kg/day, which is close to the acceptable daily intake of 0.02 mg/kg·day recommended by the WHO/FAO. To minimize unintended chemical contamination from polycarbonate bottles [18], glass drinking bottles were used. Bottles were replaced every 3 days to maintain a stable exposure concentration, and treatment was continued for 10 consecutive weeks, corresponding to two spermatogenic cycles.

### 2.3. Paternal Exposure Model and Breeding Design

Eight-week-old male mice (20–25 g) were randomly assigned to a control group receiving regular drinking water (CON) or a GLA group receiving GLA drinking water (GLA). After 10 weeks of exposure, 10 males per group were mated with unexposed 8-week-old female mice at a 1:2 male-to-female ratio. Vaginal plugs were checked the next morning, and plug rate and pregnancy rate were recorded. Offspring body weight was monitored weekly beginning at postnatal week 1, and pups were weaned at postnatal day 21. After weaning, offspring were housed by sex at 4–5 animals per cage.

### 2.4. Offspring Behavioral Assessment

Offspring were not subjected to any additional interventions and were tested at 5 weeks of age. To reduce experiment-related stress, animals were habituated to the experimenter for one week prior to formal behavioral testing. Behavioral testing was performed in both female and male offspring. Representative sample sizes used in the behavioral datasets were as follows: three-chamber test (female *n* = 42, male *n* = 32), open field test (female *n* = 42, male *n* = 32), elevated plus maze (female *n* = 30, male *n* = 30), rotarod (female *n* = 20, male *n* = 20), and Morris water maze (female *n* = 32, male *n* = 30). These sample sizes were selected with reference to the previous literature, our prior experimental experience, and the practical requirements of conducting multiple behavioral assays within the same developmental stage. Because the behavioral battery included assays with different time requirements and logistical demands, and because repeated testing of the same animals across all paradigms could introduce excessive stress, fatigue, and carryover effects, different subsets of offspring from each litter were assigned to different behavioral tests. Numbers represent animals included in the final analysis for each endpoint. Behavioral assays were performed using automated trajectory tracking systems when applicable. All apparatuses were cleaned with 70% ethanol between animals to minimize olfactory carryover.

#### 2.4.1. Three-Chamber Social Test

The three-chamber apparatus (60 cm × 35 cm × 30 cm) was divided into three equal compartments by two partitions (35 cm × 30 cm each). The test was performed according to established protocols with minor modifications [19]. Mice were placed into the arena facing the wall and habituated for 10 min. Two consecutive 10 min phases were conducted. In the sociability phase, a same-sex unfamiliar mouse (Mouse 1) was placed in a wire cage in one side chamber, and an empty wire cage was placed in the other. The interaction time with Mouse 1 versus the empty cage was recorded. In the social novelty phase, Mouse 1 remained in place, and a novel same-sex mouse (Mouse 2) was placed in the previously empty cage. The interaction time with the familiar mouse versus the novel mouse was recorded.

#### 2.4.2. Morris Water Maze Test

A circular pool (2 m diameter) was filled with water made opaque using milk, and water temperature was maintained at 18–22 °C. The Morris water maze was conducted according to established procedures for spatial learning and memory assessment with minor modifications [20]. The test included a navigation phase and a probe phase. During navigation, training lasted 3 days. Each day animals were released facing the pool wall from four starting positions, and escape latency to a hidden platform (submerged below the water surface) was recorded. On the probe day, the platform was removed. Mice were released from the start position farthest from the former platform location and tracked for 60 s to evaluate spatial memory.

#### 2.4.3. Open Field Test

Mice were placed facing the wall into a clean square arena (50 cm × 50 cm × 60 cm) and allowed to explore freely for 6 min. The open field test was carried out according to standard procedures for the assessment of locomotor activity and anxiety-related behavior, with minor modifications [21]. The floor was divided into 16 equal squares; the central four squares were defined as the center zone. Time spent in the center zone and locomotor parameters, including distance traveled, velocity, and immobility time, were quantified.

#### 2.4.4. Elevated Plus Maze Test

The elevated plus maze comprised open arms, closed arms, and a central platform. It was performed according to established procedures for anxiety-related behavior assessment, with minor modifications [22]. Mice were placed onto the central platform and allowed to explore for 5 min. Entries into each zone and time spent in open and closed arms were recorded.

#### 2.4.5. Rotarod Test

Motor coordination was assessed with an accelerating rotarod (rod diameter 3 cm). It was conducted according to standard procedures with minor modifications [23]. During testing, the rod accelerated from 5 to 25 cm/s over 120 s. Latency to fall was recorded with a maximum trial duration of 6 min. Each mouse completed three trials separated by 30 min per day for three consecutive days.

### 2.5. Brain Collection and Specimen Preparation

Based on the observed phenotype distribution, subsequent mechanistic analyses prioritized female offspring as the primary focus in this study.

#### 2.5.1. Tissue Collection for Protein Analysis

At 6 weeks of age, offspring were euthanized by CO_2_ inhalation, and whole brains were dissected and weighed, snap-frozen in liquid nitrogen, and stored at −80 °C. Fetal brains at embryonic day 14.5 (E14.5) and E17.5 were collected following euthanasia of pregnant dams and dissection of fetuses, and stored using the same procedure.

#### 2.5.2. Tissue Preparation for Histology and Immunostaining

For histology, mice were anesthetized using an isoflurane system and perfused transcardially with 4% neutral paraformaldehyde. Whole brains were removed and post-fixed in 4% paraformaldehyde at 4 °C for 12 h. Brains were then cut into 3–4 mm slabs at the optic chiasm and superior colliculus levels, fixed again in fresh 4% paraformaldehyde at 4 °C for 24 h, dehydrated through graded ethanol, cleared in xylene, embedded in paraffin, and sectioned. For E14.5 and E17.5 fetal tissues, fetal heads were fixed overnight in 4% paraformaldehyde; fetal brains were dissected, fixed in fresh paraformaldehyde for an additional 24 h, and processed for paraffin embedding and sectioning using the same workflow. Histology, immunofluorescence, and TUNEL quantification used three biological replicates per group. For image-based quantification, three non-overlapping coronal sections per animal (spaced at least 100 μm apart) were analyzed, and multiple matched fields were averaged to generate one value per animal.

#### 2.5.3. Nissl Staining and Morphometric Analysis

Paraffin sections were deparaffinized with xylene (two changes, 15 min each), rehydrated through graded ethanol (100%, 90%, 80%, 70%), and rinsed in distilled water. Nissl staining was performed according to the manufacturer’s protocol (Beyotime, Shanghai, China, C0117), and sections were mounted. Slides were digitized using a pathological slide scanner. ImageJ (v1.5.1) was used for quantitative analyses, including hippocampal area measurement, prefrontal cortex thickness measurement, and neuronal counting within regions of interest.

#### 2.5.4. Golgi Staining and Dendritic Analysis

Golgi staining was performed using a Golgi Cox protocol (Servicebio, Wuhan, China) with the following key steps: immersion in Golgi Cox solution for 48 h in the dark, rinsing, graded dehydration, ammonium hydroxide treatment, sodium thiosulfate treatment, further dehydration, clearing in xylene or toluene, and mounting. Stained sections were digitized in bright-field mode using a tissue section digital scanner (LG-S80, Servicebio, Wuhan, China), and scanned images were viewed and exported using Saiviewer digital slide viewing software (v2.2.2, Servicebio, Wuhan, China).

For dendritic morphology analysis, female offspring from three independent litters were included in each group (*n* = 3 animals per group). In the dentate gyrus of the hippocampus and prefrontal cortex, neurons with a clearly identifiable soma, complete and well-impregnated dendritic arborization, and minimal overlap with neighboring stained neurons were sampled from three non-overlapping fields for each animal, with approximately five neurons analyzed per field. Thus, approximately 15 neurons per animal were analyzed in each region. Sholl analysis was performed in ImageJ using concentric circles with 10 μm ring spacing. For dendritic spine analysis, spines were quantified on secondary or tertiary dendritic branches, and each analyzed dendritic segment was at least 10 μm in uninterrupted length. For statistical analysis, when multiple neurons were analyzed from the same animal, neuron-level measurements were averaged to generate one value per animal, and the animal was used as the unit for statistical comparison.

#### 2.5.5. Immunofluorescence Staining

Paraffin sections were deparaffinized and rehydrated, followed by antigen retrieval in sodium citrate buffer using microwave pulse heating and passive cooling to room temperature. Sections were blocked for 1–2 h and incubated with primary antibodies overnight at 4 °C. After PBS washes, sections were incubated with fluorophore-conjugated secondary antibodies for 2 h at room temperature, counterstained with DAPI (Abcam, Cambridge, UK, ab104139), and mounted. Markers included GFAP for astrocytes (Abcam, Cambridge, UK, ab7260), IBA1 for microglia (Abcam, Cambridge, UK, ab178846), VGLUT1 for excitatory neurons (Abcam, Cambridge, UK, ab227805), and GAD1 for inhibitory neurons (Abcam, Cambridge, UK, ab26116) from dentate gyrus of the hippocampus and prefrontal cortex within each experiment. Images were acquired using a Zeiss LSM710 confocal microscope (Carl Zeiss, Oberkochen, Germany) with constant exposure time, offset and gain, and quantification was performed in ImageJ. For each animal, three non-overlapping coronal sections spaced at least 100 μm apart were analyzed, and at least three randomly selected non-overlapping fields were acquired from each region of interest in each section. Fluorescence signals were quantified using identical acquisition and analysis settings, and field-level measurements were averaged to generate one value per animal from different litters for statistical comparison.

#### 2.5.6. TUNEL Assay

Apoptosis in brain sections was evaluated using the TUNEL BrightRed Apoptosis Detection Kit according to the manufacturer’s instructions (Vazyme, Nanjing, China, A113). For hippocampal and prefrontal cortex analyses, fields were selected according to stage-appropriate anatomical landmarks at E14.5, E17.5, and 5 weeks. Because brain cytoarchitecture differs substantially across these developmental stages, the representative field shown was not identical at all time points. Images were obtained using a Zeiss LSM710 confocal microscope. For each animal, three non-overlapping coronal sections spaced at least 100 μm apart were analyzed, and at least three non-overlapping fields were acquired for each region in each section. All acquired images were included in the final quantification. Quantification was performed in ImageJ as the percentage of TUNEL-positive cells within a standardized field of view. Field-level measurements were averaged to generate one value per animal for statistical comparison.

#### 2.5.7. Western Blotting

For protein extraction, the hippocampus and prefrontal cortex were dissected on ice from brains stored at −80 °C, with one animal contributing one sample. Approximately 0.1 g tissue was homogenized in 300–500 μL RIPA lysis buffer containing 1% protease inhibitor (Beyotime, P0013 and P1005), followed by centrifugation at 4 °C (12,000 rpm, 15 min). Protein concentration was determined by a BCA assay. Samples were prepared to load 80 μg total protein per lane, an empirically optimized working condition in our laboratory, and denatured at 100 °C for 10 min. Proteins were separated by SDS-PAGE and transferred to PVDF membranes. Membranes were blocked in 5% non-fat milk for 1 h and incubated with primary antibodies (typically 1:1000) overnight at 4 °C, followed by HRP-conjugated secondary antibodies (typically 1:1000) for 1 h at room temperature. Markers included Caspase-3 (Affinity Biosciences, Liyang, China, AF6311), Bcl-2 (Cell Signaling Technology, Danvers, MA, USA, 3498), IL-1β (Cell Signaling Technology, Danvers, MA, USA, 12242), IL-10 (Affinity Biosciences, Liyang, China, DF6894), GAPDH (Cell Signaling Technology, Danvers, MA, USA, 97166), and α/β-Tubulin (Cell Signaling Technology, Danvers, MA, USA, 2148). Bands were visualized using chemiluminescence and quantified by ImageJ (v1.5.1).

#### 2.5.8. Statistical Analysis

All quantitative analyses were performed using GraphPad Prism (version 7.0.0). Data are presented as mean ± SEM. For two-group comparisons, Student’s *t*-test was used when data met assumptions of independence, normality, and homogeneity of variance; otherwise, the Wilcoxon rank-sum test was applied. For multi-group continuous data, one-way ANOVA was used when assumptions were met. Categorical variables were analyzed using chi-square tests. For behavioral endpoints, offspring values were analyzed using a mixed-effects framework that accounted for litter as a clustering factor. Because offspring from the same litter are not statistically independent, litter-related variation was considered in the inferential analysis, while the reported offspring numbers indicate the animals tested in each assay. A two-sided *p* value < 0.05 was considered statistically significant. In the figures, significance levels are denoted as follows: NS: no significant difference; *: *p* < 0.05; **: *p* < 0.01; ***: *p* < 0.001.

## 3. Results

### 3.1. Offspring Growth and Neurobehavior

We first examined whether paternal preconception GLA exposure affected general growth of F1 offspring. Body weight from postnatal week 1 to week 5 was similar between CON and GLA offspring in both females and males. Body length measured at 5 weeks also showed no difference between groups (Figure S1). These findings suggest that paternal GLA exposure did not affect overall postnatal growth and provide a stable baseline for interpreting neurobehavioral outcomes.

**Figure 1:**
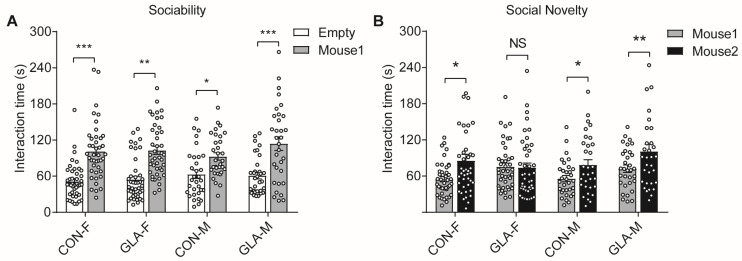
**Three-chamber test.** (**A**) Sociability phase. (**B**) Social novelty phase. CON-F and CON-M indicate offspring from control groups, and GLA-F and GLA-M indicate offspring from GLA exposed groups. Bars represent mean ± SEM and dots represent individual pups. NS, not significant; * *p* < 0.05; ** *p* < 0.01; *** *p* < 0.001.

We then assessed social behavior using the three-chamber test. In the sociability phase, both CON and GLA offspring of each sex spent more time with the stranger mouse than with the empty cage, indicating the normal social approach (Figure 1A). In the social novelty phase, CON females spent more time with the novel mouse than with the familiar mouse, while GLA females did not show this preference (Figure 1B). In males, both CON and GLA offspring preferred the novel mouse over the familiar mouse. These results indicate that paternal GLA exposure reduced social novelty preference in female offspring but did not affect basic sociability. It suggests that the change was more evident in the novelty or memory component of the task rather than in the initial social approach component.

**Figure 2:**
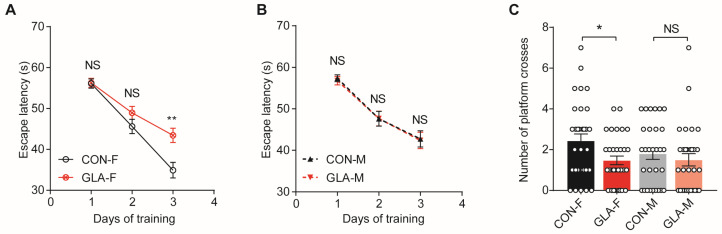
**Morris water maze test.** (**A**) Escape latency during navigation training in female offspring. (**B**) Escape latency during acquisition training in male offspring. (**C**) Probe test performance shown as number of platform area crossings for female and male offspring. CON-F and CON-M indicate offspring from control groups, and GLA-F and GLA-M indicate offspring from GLA exposed groups. Line and bar plots show mean ± SEM with dots indicating individual pups. NS, not significant; * *p* < 0.05; ** *p* < 0.01.

Next, we tested spatial learning and memory in the Morris water maze. During training, female offspring in the GLA group required more time to find the hidden platform than CON females (*p* < 0.01), while males showed no clear group difference (Figure 2A,B). In the probe test, female GLA offspring crossed the former platform location fewer times than CON females (*p* < 0.05), whereas males showed no difference between groups (Figure 2C). These data indicate impaired spatial memory in female offspring after paternal GLA exposure. Together with the social novelty results, the behavioral phenotype was mainly observed in females and was centered on memory-related domains at 5 weeks of age.

To determine whether paternal GLA exposure altered activity, anxiety-like behavior, or motor ability, we performed additional tests. In the open field test, time spent in the center area was similar between groups in both sexes (Figure S2A). In the elevated plus maze test, time in the open arms did not differ between groups (Figure S2B). Rotarod performance was also similar between groups (Figure S2C). These negative results help reduce the possibility that the memory-related differences were driven by altered locomotion, anxiety level, or motor coordination.

### 3.2. Brain Morphology and Histology

We next examined whether paternal GLA exposure caused obvious changes in brain morphology at 5 weeks. Brain appearance was similar between groups. Brain weight relative to body weight and the brain length to width ratio did not differ between CON and GLA offspring (Figure S3A–C). Measurements of cortical thickness and hippocampal size also showed no difference between groups (Figure S3D,E). Nissl staining did not show clear disruption of general brain structure (Figure S3F). These data indicate no obvious gross brain changes in offspring after paternal GLA exposure. Thus, the observed behavioral differences were not accompanied by large-scale changes in brain size or overall cytoarchitecture detectable by these measures.

**Figure 3:**
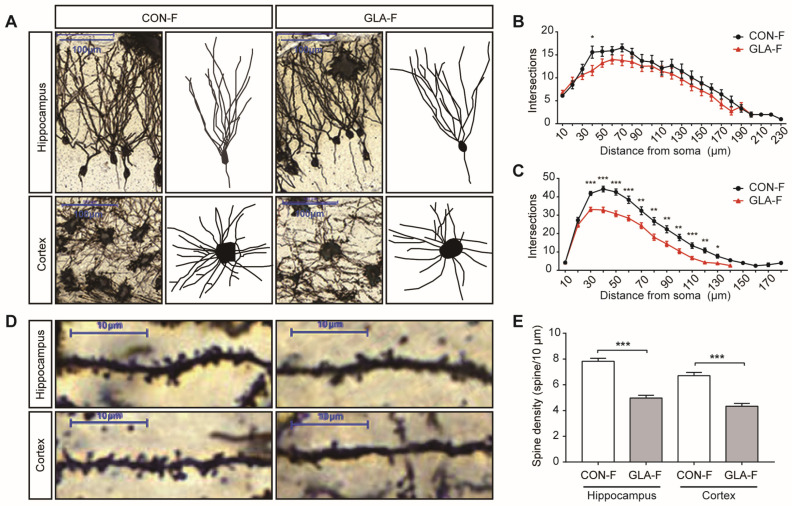
**Golgi analysis.** (**A**) Representative Golgi stained neurons in hippocampus and prefrontal cortex from CON-F and GLA-F offspring. Scale bar, 100 μm. (**B**) Sholl analysis of dendritic complexity in dentate gyrus granule neurons in the hippocampus. (**C**) Sholl analysis of dendritic complexity in prefrontal cortical neurons. (**D**) Representative dendritic segments used for spine analysis in hippocampus and prefrontal cortex. Scale bar, 10 μm. (**E**) Quantification of dendritic spine density (spines per 10 μm) in hippocampus and prefrontal cortex. For Golgi analysis, female offspring from three independent litters were included in each group (*n* = 3 animals per group), and approximately 15 neurons per animal were analyzed in each region. Neuron-level measurements were averaged within each animal for statistical comparison. Bars represent mean ± SEM. * *p* < 0.05; ** *p* < 0.01; *** *p* < 0.001.

Because memory-related behaviors were affected in females, we then examined neuronal structure in female offspring using Golgi staining. Compared with CON, dentate gyrus granule neurons in the hippocampus and neurons in the prefrontal cortex from the GLA group showed fewer dendritic branches (Figure 3A). Sholl analysis confirmed reduced dendritic complexity in both regions, with a slightly larger change in the prefrontal cortex (Figure 3B,C). Spine density was also lower in both the hippocampus and prefrontal cortex in the GLA group than in controls (Figure 3D,E, *p* < 0.001 for both). These results indicate impaired neuronal structural features in key brain regions in female offspring after paternal GLA exposure. Reduced dendritic complexity and lower spine density are consistent with weaker synaptic structural support in these regions, providing a structural correlate for altered memory-related behavior.

### 3.3. Glia and Neuronal Markers Detection

**Figure 4:**
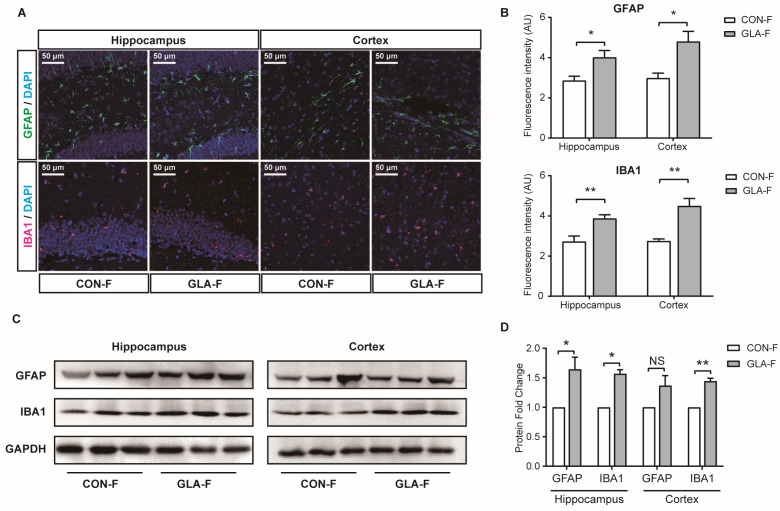
**GFAP and IBA1.** (**A**) Representative immunofluorescence images of GFAP (green) and IBA1 (magenta) with DAPI counterstain (blue) in the dentate gyrus of the hippocampus and in the prefrontal cortex from CON-F and GLA-F offspring. Scale bar, 50 μm. (**B**) Quantification of fluorescence intensity for GFAP and IBA1. For immunofluorescence quantification, three non-overlapping coronal sections per animal and at least three non-overlapping fields per region per section were analyzed, and field-level measurements were averaged within each animal for statistical comparison. (**C**) Representative immunoblots of GFAP and IBA1 in hippocampus and prefrontal cortex, with GAPDH as a loading control. (**D**) Quantification of immunoblot band intensity shown as protein fold change relative to CON-F. Bars represent mean ± SEM. NS, not significant; * *p* < 0.05; ** *p* < 0.01.

We next assessed glial markers in female offspring. Immunofluorescence showed higher GFAP and IBA1 signals in the dentate gyrus region of the hippocampus (*p* < 0.05 for both) and in the prefrontal cortex (*p* < 0.01) in the GLA group compared with CON (Figure 4A,B). Western blotting confirmed increased GFAP and IBA1 in the hippocampus (*p* < 0.05 for both). In the prefrontal cortex, IBA1 was increased (*p* < 0.01), while GFAP did not show a clear difference by Western blot (Figure 4C,D). These results indicate increased astrocyte and microglia markers in female offspring after paternal GLA exposure.

We also measured markers related to excitatory and inhibitory neurons. In immunofluorescence, GAD1 was higher in both the hippocampus and prefrontal cortex in the GLA group (*p* < 0.05 for both), while vGLUT1 was lower in the hippocampus (*p* < 0.05) (Figure S4A,B). Western blotting showed lower vGLUT1 in the hippocampus (*p* < 0.05) and higher GAD1 in the prefrontal cortex (*p* < 0.01) (Figure S4C,D). These results suggest that paternal GLA exposure is associated with changes in neuronal marker expression, with different patterns across brain regions. In particular, reduced hippocampal vGLUT1 together with increased GAD1 signals is consistent with a shift in molecular markers linked to excitatory and inhibitory neuronal features in regions relevant to memory processing.

### 3.4. Apoptosis and Cytokine Marker Detection

**Figure 5:**
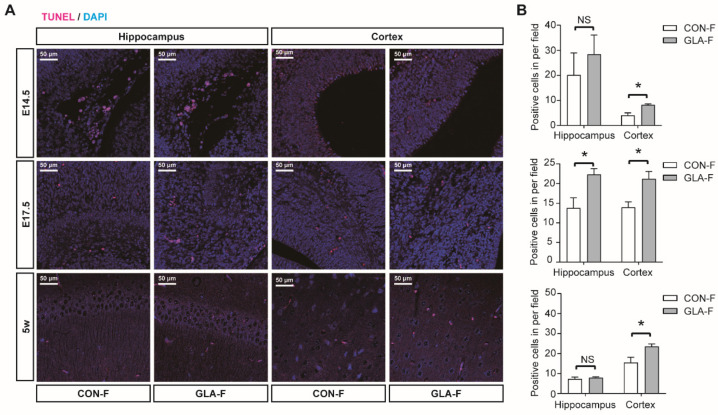
**TUNEL staining.** (**A**) Representative TUNEL staining (magenta) with DAPI counterstain (blue) in stage-matched hippocampal fields and prefrontal cortex from CON-F and GLA-F offspring at embryonic day 14.5 (E14.5), embryonic day 17.5 (E17.5), and postnatal week 5. Scale bar, 50 μm. (**B**) Quantification of TUNEL-positive cells per field for each region and time point. For TUNEL quantification, three non-overlapping coronal sections per animal and at least three non-overlapping fields per region per section were analyzed, and field-level measurements were averaged within each animal for statistical comparison. Bars represent mean ± SEM. NS, not significant; * *p* < 0.05.

To examine cell death during development, we performed TUNEL staining at E14.5, E17.5, and 5 weeks in female offspring brain tissue. For the hippocampus and prefrontal cortex, representative fields were selected according to stage-appropriate anatomical landmarks at each developmental stage, and quantitative analysis was performed on matched fields across groups within each stage. At E14.5, TUNEL-positive cells were increased in the prefrontal cortex in the GLA group (*p* < 0.05). At E17.5, TUNEL-positive cells were increased in both the hippocampus and prefrontal cortex (*p* < 0.05 for both). At 5 weeks, TUNEL positivity remained higher in the prefrontal cortex (*p* < 0.05), while the hippocampus showed no clear difference (Figure 5A,B). Across time points, the prefrontal cortex showed a more sustained apoptosis signal, whereas hippocampal differences were most evident at E17.5. These data indicate increased apoptosis signals during embryonic development, with persistence into the juvenile prefrontal cortex.

We next measured apoptosis- and cytokine-related proteins in the hippocampus and prefrontal cortex by Western blotting. In the hippocampus, Bcl-2 was reduced (*p* < 0.01), IL-1β was increased (*p* < 0.05), and IL-10 was reduced (*p* < 0.01) in the GLA group compared with CON. In the prefrontal cortex, Caspase-3 was increased (*p* < 0.001) and IL-10 was reduced (*p* < 0.05), while changes in Bcl-2 and IL-1β were less evident (Figure S5). Combining with the increased glial marker expression observed in the hippocampus and prefrontal cortex, increased IL-1β together with reduced IL-10 in the hippocampus is consistent with an altered neuroimmune-related profile and may suggest a tendency toward a more pro-inflammatory state. The prefrontal cortex showed reduced IL-10 along with increased Caspase-3, consistent with enhanced apoptosis-related signaling in this region. Together, these results indicate increased apoptosis and an altered cytokine profile in female offspring after paternal GLA exposure, with a stronger and more consistent pattern in the prefrontal cortex.

## 4. Discussion

Across endpoints, our findings fit with a broader idea in the paternal exposure literature. Conditions in fathers before mating can shape offspring brain and behavior, even when offspring show normal growth. Several paternal exposure studies report behavioral and brain molecular changes in offspring without clear signs of general toxicity, which supports the view that paternal factors can influence neurodevelopment in a meaningful way [24,25,26,27]. In this context, the present study contributes something that has been largely missing from the GLA literature: direct phenotype-level evidence that paternal preconception exposure, under natural mating conditions, is associated with offspring neurobehavioral and neuropathological alterations.

A clear point from our behavioral results is the selective memory-related changes. In the three-chamber test, the social approach was normal, but the social novelty preference was reduced in females. In the Morris water maze test, females showed poorer learning and memory, while open field, elevated plus maze, and rotarod did not show clear differences. This pattern matters because it rules out simple explanations such as reduced movement or increased anxiety. In other developmental and intergenerational studies, the results are more consistent with a cognitive effect than with a general performance problem [28]. The female-biased pattern in our study is in line with reports from developmental models showing that biological sex can influence susceptibility and the timing of detectable cognitive outcomes [29]. It is important to note that the mechanistic endpoints in the present study (Golgi morphology, glial markers, TUNEL, and cytokine measurements) were assessed only in female offspring as the behavioral phenotype was observed predominantly in this sex. With this limitation in mind, one possible explanation is that neural features show sex differences across development, including differences in morphology and inflammatory gene or cytokine expression in brain regions relevant to cognition [30,31]. In addition, sex differences have been described in prefrontal cortex microglia during development, which may influence how inflammatory signals are expressed and how circuits mature in this region [32,33]. Direct comparisons of these endpoints in female and male offspring will be required in further study to determine whether such biological sex differences contribute to the selective vulnerability observed here.

In addition to the behavioral findings, our Golgi analyses revealed reduced dendritic complexity and decreased dendritic spine density in the hippocampal dentate gyrus and prefrontal cortex of female offspring. These findings are important because dendritic arborization influences how neurons receive and integrate afferent inputs, whereas dendritic spines constitute the principal structural substrate of most excitatory synapses [34]. Therefore, the combined reduction in dendritic branching and spine density suggests impaired neuronal structural maturation and weaker synaptic structural support in two brain regions that are critical for novelty-related processing and spatial memory [35,36]. In this context, the morphological changes observed here provide a plausible anatomical correlate for the selective memory-related behavioral deficits detected in female offspring.

The glial and immune-related changes in the hippocampus and prefrontal cortex provide a plausible biological context for the memory-related behavioral findings. In our study, GFAP and IBA1 were higher in both regions, which indicates increased astrocyte and microglia marker expression. In developmental and intergenerational research, changes in glial state and immune signaling are often discussed as factors that can affect synapse development and later cognitive function, particularly when cytokine regulation shifts toward a more inflammatory pattern [37]. Our cytokine results showed an increase in IL-1β together with a decrease in IL-10. Although this limited cytokine panel does not provide a comprehensive definition of the inflammatory status, this pattern is consistent with altered inflammatory regulation and may suggest a tendency toward stronger pro-inflammatory signaling with weaker anti-inflammatory counter-regulation. In parallel, apoptosis-related readouts were also higher, which is consistent with cell stress and cell death-related signaling in the developing and juvenile brain. This set of findings does not demonstrate that inflammation causes the behavioral phenotype, but it provides supportive evidence that paternal preconception GLA exposure is associated with neuroimmune-related disturbance in brain regions relevant to memory function, which matches reports that developmental immune imbalance is linked with persistent changes in learning and memory [29]. This regional pattern suggests that memory and social novelty depend on multiple brain regions that differ in developmental timing and local immune regulation, so one exposure like GLA can alter molecular signatures across the network while contributing to a related behavioral outcome.

Although our study does not include germline measurements, it is still reasonable to discuss how paternal exposure might influence the next generation because the study is built around a paternal preconception design. Reviews emphasize that sperm carry regulatory information beyond DNA sequence, including chromatin features and RNA species that can affect gene regulation soon after fertilization [38,39]. Other reviews summarize evidence that paternal exposures can change sperm small RNAs and that these RNAs can influence offspring phenotypes in experimental systems [40]. Sperm content is also not fixed at the end of spermatogenesis. Remodeling during epididymal maturation has been described as a period when sperm regulatory cargo can be modified, which makes the preconception period biologically relevant [41]. For GLA specifically, our previous studies showed that paternal GLA exposure was associated with altered sperm epigenetic information, including abnormal DNA methylation- and histone modification-related patterns, and that these changes were concordant with transcriptomic alterations in preimplantation embryos. The affected pathways included functions related to nervous system development and immune/inflammatory regulation [15,16]. These prior findings provide a biologically plausible framework for the present results, in which paternal preconception GLA exposure was associated with memory-related behavioral deficits and convergent structural, glial, cytokine-related, and apoptosis-related alterations in the offspring brain. In this sense, the current study extends our previous molecular observations by showing that the consequences of paternal GLA exposure may not remain limited to epigenetic or transcriptomic abnormalities in the germline and early embryo but may also manifest as detectable neurodevelopmental and neurobehavioral alterations in the offspring. Thus, more direct experiments are needed to test whether the intergenerational effects come mainly from changes carried by sperm or from other paternal influences before conception.

The present work has several strengths that support the interpretation of the findings. A major strength is that we combined behavioral testing with multiple brain readouts across regions and developmental windows, which allowed us to identify a consistent pattern linking memory-related outcomes with glial, cytokine, and apoptosis-related signals. The limitations of the present work also point to clear next steps. First, our histological and immunostaining analyses were performed with three biological replicates. Although multiple matched sections and fields were quantified for each animal and the resulting values were averaged at the animal level, these image-based data should be interpreted as supportive rather than standalone evidence. Second, our neuroimmune evidence is based on marker expression and cytokine levels. This is a reasonable starting point to show that brain immune-related signals change in a consistent direction across regions. A possible next step is to add more functional and cell type resolved measures and to test whether changing neuroinflammation or microglial state can modify the phenotype. Third, only a single paternal GLA dose was tested in the present study. Because currently available human biomonitoring data for GLA remain limited, especially for direct evaluation of male and occupational internal exposure, it is difficult to directly relate this experimental dose to real-world population exposure. In addition, the present design does not allow assessment of a dose–response relationship or definition of a toxicological threshold for paternal effects. Thus, the dose used here should be interpreted as a relatively low experimental paternal exposure condition selected on the basis of currently available toxicological information and our previous work, rather than as a defined threshold for human risk. Fourth, we focused on one juvenile age window. This is a practical choice for detecting early neurodevelopmental outcomes, but it does not tell us whether the phenotype persists or changes later. Follow-up into adolescence and adulthood would clarify whether behavior and brain signatures remain stable, recover, or evolve. Lastly, the female-biased pattern is an important observation, and sex-dependent effects have been reported in developmental immune and inflammatory models. A systematic comparison across sexes, using the same behavioral and brain measures across multiple ages, will help clarify whether the difference reflects developmental timing, neuroimmune responses, or other factors.

## 5. Conclusions

Paternal preconception exposure to GLA was associated with selective memory-related behavioral deficits in female offspring within a natural-mating design. These behavioral changes were accompanied by convergent alterations in dendritic structure, glial markers, cytokine-related signaling, and apoptosis-related readouts in the hippocampus and prefrontal cortex. Together, these findings support the view that paternal exposure should be considered in developmental neurotoxicity assessment. At the same time, because the present study used a single paternal dose and a natural-mating model, future studies will be needed to define dose-response relationships, clarify persistence across developmental stages, and more directly investigate the mechanisms by which paternal exposure influences offspring neurodevelopment.

## Data Availability

The original contributions presented in this study are included in the article/Supplementary Material. Further inquiries can be directed to the corresponding authors.

## Supplementary Materials

The following supporting information can be downloaded at: https://www.mdpi.com/article/10.3390/toxics14050396/s1, Figure S1: Offspring growth.; Figure S2. Baseline behavior; Figure S3. Brain morphology; Figure S4. GAD1 and vGLUT1; Figure S5. Cytokines and apoptosis.

## References

[1] Filippi I., Bonansea R.I., Butinof M., Fernández R.A., Llorca M., Farré M., Muñoz S.E., Amé M.V. (2023). First Report of the Joint Exposure to Glyphosate and Glufosinate of a Male Population in the Province of Córdoba (Argentina). Toxics.

[2] Ashley-Martin J., Huang R., MacPherson S., Brion O., Owen J., Gaudreau E., Bienvenu J.F., Fisher M., Borghese M.M., Bouchard M.F. (2023). Urinary concentrations and determinants of glyphosate and glufosinate in pregnant Canadian participants in the MIREC study. Environ. Res..

[3] Dong T., Guan Q., Hu W., Zhang M., Zhang Y., Chen M., Wang X., Xia Y. (2020). Prenatal exposure to glufosinate ammonium disturbs gut microbiome and induces behavioral abnormalities in mice. J. Hazard. Mater..

[4] Meme S., Calas A.G., Montécot C., Richard O., Gautier H., Gefflaut T., Doan B.T., Même W., Pichon J., Beloeil J.C. (2009). MRI characterization of structural mouse brain changes in response to chronic exposure to the glufosinate ammonium herbicide. Toxicol. Sci..

[5] Calas A.G., Perche O., Richard O., Perche A., Pâris A., Lauga F., Herzine A., Palomo J., Ardourel M.Y., Menuet A. (2016). Characterization of seizures induced by acute exposure to an organophosphate herbicide, glufosinate-ammonium. Neuroreport.

[6] Laugeray A., Herzine A., Perche O., Hébert B., Aguillon-Naury M., Richard O., Menuet A., Mazaud-Guittot S., Lesné L., Briault S. (2014). Pre- and postnatal exposure to low dose glufosinate ammonium induces autism-like phenotypes in mice. Front. Behav. Neurosci..

[7] Herzine A., Laugeray A., Feat J., Menuet A., Quesniaux V., Richard O., Pichon J., Montécot-Dubourg C., Perche O., Mortaud S. (2016). Perinatal Exposure to Glufosinate Ammonium Herbicide Impairs Neurogenesis and Neuroblast Migration through Cytoskeleton Destabilization. Front. Cell. Neurosci..

[8] Feat-Vetel J., Larrigaldie V., Meyer-Dilhet G., Herzine A., Mougin C., Laugeray A., Gefflaut T., Richard O., Quesniaux V., Montécot-Dubourg C. (2018). Multiple effects of the herbicide glufosinate-ammonium and its main metabolite on neural stem cells from the subventricular zone of newborn mice. Neurotoxicology.

[9] Watanabe T. (1997). Apoptosis induced by glufosinate ammonium in the neuroepithelium of developing mouse embryos in culture. Neurosci. Lett..

[10] Maillet I., Perche O., Pâris A., Richard O., Gombault A., Herzine A., Pichon J., Huaux F., Mortaud S., Ryffel B. (2016). Glufosinate aerogenic exposure induces glutamate and IL-1 receptor dependent lung inflammation. Clin. Sci..

[11] Oummadi A., Menuet A., Méresse S., Laugeray A., Guillemin G., Mortaud S. (2023). The herbicides glyphosate and glufosinate and the cyanotoxin β-N-methylamino-l-alanine induce long-term motor disorders following postnatal exposure: The importance of prior asymptomatic maternal inflammatory sensitization. Front. Neurosci..

[12] Day J., Savani S., Krempley B.D., Nguyen M., Kitlinska J.B. (2016). Influence of paternal preconception exposures on their offspring: Through epigenetics to phenotype. Am. J. Stem Cells.

[13] Yeshurun S., Rogers J., Short A.K., Renoir T., Pang T.Y., Hannan A.J. (2017). Elevated paternal glucocorticoid exposure modifies memory retention in female offspring. Psychoneuroendocrinology.

[14] Goldberg L.R., Zeid D., Kutlu M.G., Cole R.D., Lallai V., Sebastian A., Albert I., Fowler C.D., Parikh V., Gould T.J. (2021). Paternal nicotine enhances fear memory, reduces nicotine administration, and alters hippocampal genetic and neural function in offspring. Addict. Biol..

[15] Ma X., Wang B., Li Z., Ding X., Wen Y., Shan W., Hu W., Wang X., Xia Y. (2022). Effects of glufosinate-ammonium on male reproductive health: Focus on epigenome and transcriptome in mouse sperm. Chemosphere.

[16] Ma X., Fan Y., Xiao W., Ding X., Hu W., Xia Y. (2021). Glufosinate-Ammonium Induced Aberrant Histone Modifications in Mouse Sperm Are Concordant With Transcriptome in Preimplantation Embryos. Front. Physiol..

[17] Nair A.B., Jacob S. (2016). A simple practice guide for dose conversion between animals and human. J. Basic. Clin. Pharm..

[18] vom Saal F.S., Hughes C. (2005). An extensive new literature concerning low-dose effects of bisphenol A shows the need for a new risk assessment. Environ. Health Perspect..

[19] Yang M., Silverman J.L., Crawley J.N. (2011). Automated three-chambered social approach task for mice. Curr. Protoc. Neurosci..

[20] Vorhees C.V., Williams M.T. (2006). Morris water maze: Procedures for assessing spatial and related forms of learning and memory. Nat. Protoc..

[21] Kraeuter A.K., Guest P.C., Sarnyai Z. (2019). The Open Field Test for Measuring Locomotor Activity and Anxiety-Like Behavior. Methods Mol. Biol..

[22] Walf A.A., Frye C.A. (2007). The use of the elevated plus maze as an assay of anxiety-related behavior in rodents. Nat. Protoc..

[23] Deacon R.M. (2013). Measuring motor coordination in mice. J. Vis. Exp..

[24] Chan J.C., Nugent B.M., Bale T.L. (2018). Parental Advisory: Maternal and Paternal Stress Can Impact Offspring Neurodevelopment. Biol. Psychiatry.

[25] Yaw A.M., Prosser R.A., Jones P.C., Garcia B.J., Jacobson D.A., Glass J.D. (2019). Epigenetic effects of paternal cocaine on reward stimulus behavior and accumbens gene expression in mice. Behav. Brain Res..

[26] Ju L.S., Zhu J., Morey T.E., Gravenstein N., Seubert C.N., Setlow B., Martynyuk A.E. (2024). Neurobehavioral Abnormalities in Offspring of Young Adult Male Rats With a History of Traumatic Brain Injury. J. Neurotrauma.

[27] Hing B., Taylor R., Eliasen S., Stevens H.E. (2025). Parental preconceptual α-cypermethrin exposure alters embryonic brain transcriptomics in mice: Implications for autism spectrum disorder and stress vulnerability. Neurotoxicology.

[28] Raymann S., Schalbetter S.M., Schaer R., Bernhardt A.C., Mueller F.S., Meyer U., Weber-Stadlbauer U. (2023). Late prenatal immune activation in mice induces transgenerational effects via the maternal and paternal lineages. Cereb. Cortex.

[29] Zhang Z.Z., Chen J., Luo B.L., Ni M.Z., Liu X., Zeng L.P., Yang Q.G., Wang F., Chen G.H. (2022). Maternal inflammation induces spatial learning and memory impairment in the F1 and F2 generations of mice via sex-specific epigenetic mechanisms. Brain Res. Bull..

[30] Osborne B.F., Turano A., Schwarz J.M. (2018). Sex Differences in the Neuroimmune System. Curr. Opin. Behav. Sci..

[31] Schwarz J.M., Sholar P.W., Bilbo S.D. (2012). Sex differences in microglial colonization of the developing rat brain. J. Neurochem..

[32] do Prado C.H., Narahari T., Holland F.H., Lee H.N., Murthy S.K., Brenhouse H.C. (2016). Effects of early adolescent environmental enrichment on cognitive dysfunction, prefrontal cortex development, and inflammatory cytokines after early life stress. Dev. Psychobiol..

[33] Gildawie K.R., Orso R., Peterzell S., Thompson V., Brenhouse H.C. (2020). Sex differences in prefrontal cortex microglia morphology: Impact of a two-hit model of adversity throughout development. Neurosci. Lett..

[34] Runge K., Cardoso C., de Chevigny A. (2020). Dendritic Spine Plasticity: Function and Mechanisms. Front. Synaptic Neurosci..

[35] Hainmueller T., Bartos M. (2020). Dentate gyrus circuits for encoding, retrieval and discrimination of episodic memories. Nat. Rev. Neurosci..

[36] Euston D.R., Gruber A.J., McNaughton B.L. (2012). The role of medial prefrontal cortex in memory and decision making. Neuron.

[37] Lammert C.R., Frost E.L., Bolte A.C., Paysour M.J., Shaw M.E., Bellinger C.E., Weigel T.K., Zunder E.R., Lukens J.R. (2018). Cutting Edge: Critical Roles for Microbiota-Mediated Regulation of the Immune System in a Prenatal Immune Activation Model of Autism. J. Immunol..

[38] O’Doherty A.M., McGettigan P.A. (2015). Epigenetic processes in the male germline. Reprod. Fertil. Dev..

[39] Zheng X., Li Z., Wang G., Wang H., Zhou Y., Zhao X., Cheng C.Y., Qiao Y., Sun F. (2021). Sperm epigenetic alterations contribute to inter- and transgenerational effects of paternal exposure to long-term psychological stress via evading offspring embryonic reprogramming. Cell Discov..

[40] Klastrup L.K., Bak S.T., Nielsen A.L. (2019). The influence of paternal diet on sncRNA-mediated epigenetic inheritance. Mol. Genet. Genom..

[41] Bedi Y.S., Roach A.N., Thomas K.N., Mehta N.A., Golding M.C. (2022). Chromatin alterations during the epididymal maturation of mouse sperm refine the paternally inherited epigenome. Epigenetics Chromatin.

