# Supplementary material for: Paternal Glufosinate Ammonium Exposure Leads to Memory Dysfunction in Offspring Mice
Source: Toxics. 2026 May 6;14(5):396. doi: 10.3390/toxics14050396 (PMC13211454; doi:10.3390/toxics14050396)
Supplement: Supplementary file 1 [file toxics-14-00396-s001.zip › toxics-4232223-supplementary.pdf]

Paternal glufosinate-ammonium exposure leads to memory dysfunction in offspring mice

Zhilu Pei [1,2,†], Dayu Hu [1,2,†], Jie Sun [3,4,*] and Weiyue Hu [1,2,*]

[1] Key Laboratory of Modern Toxicology of Ministry of Education, School of Public Health, Nanjing Medical University, No.101 Longmian Road, Nanjing 211166, China; peizhilu@stu.njmu.edu.cn (Z.P.); hudayuu@163.com (D.H.)

[2] Department of Nutrition and Food Safety, School of Public Health, Nanjing Medical University,
Nanjing 211166, China

[3] Department of Endocrinology, Endocrine and Metabolic Disease Medical Center, Nanjing Drum Tower Hospital, Affiliated Hospital of Medical School, No.22 Hankou Road, Nanjing 210093, China

[4] Branch of National Clinical Research Centre for Metabolic Diseases, Nanjing 210093, China

† These authors contributed equally to this work and they should be regarded as joint first authors.

* Correspondence: sunjie19891104@126.com (J.S.); weiyuehu@njmu.edu.cn (W.H.); Tel.: +86-25-68182474 (J.S.); +86-25-86868452 (W.H.)

Figure legends

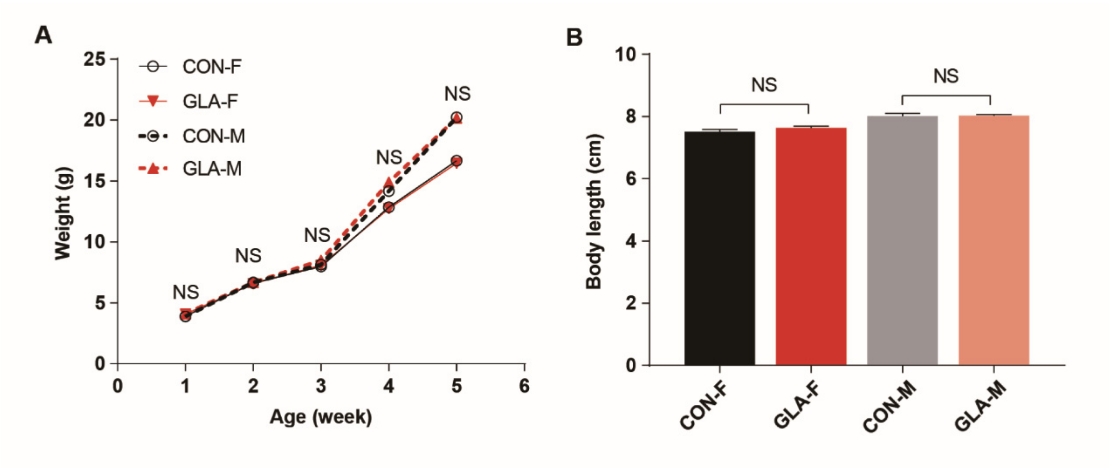

Figure S1. Offspring growth. (A) Body weight from postnatal week 1 to week 5 in female and male offspring. (B) Body length at 5 weeks. CON-F and CON-M indicate offspring from control groups, and GLA-F and GLA-M indicate offspring from GLA exposed groups. Data are mean ± SEM. NS, not significant.

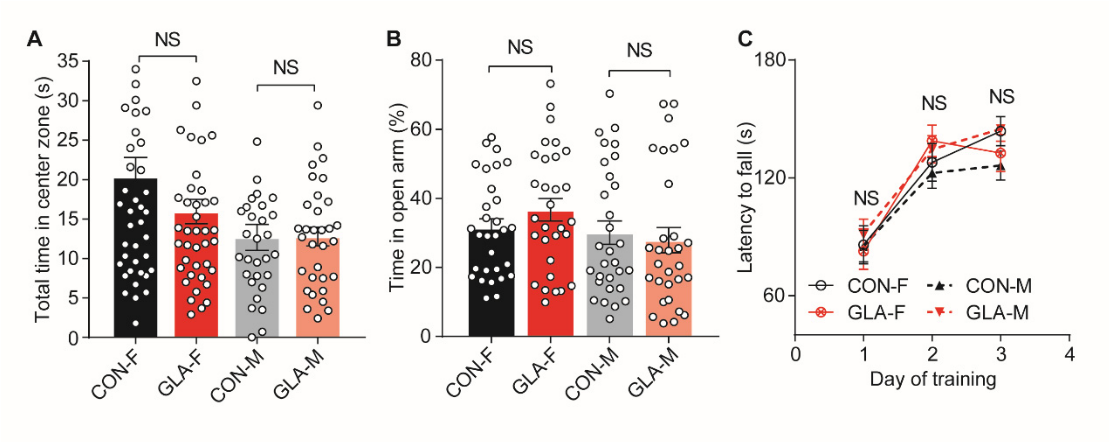

Figure S2. Baseline behavior. (A) Open field test shown as total time spent in the center zone. (B) Elevated plus maze shown as time spent in open arms. (C) Rotarod performance shown as latency to fall across training days. CON-F and CON-M indicate offspring from control groups, and GLA-F and GLA-M indicate offspring from GLA exposed groups. Bars and lines plots show mean ± SEM with dots indicating individual pups. NS, not significant.

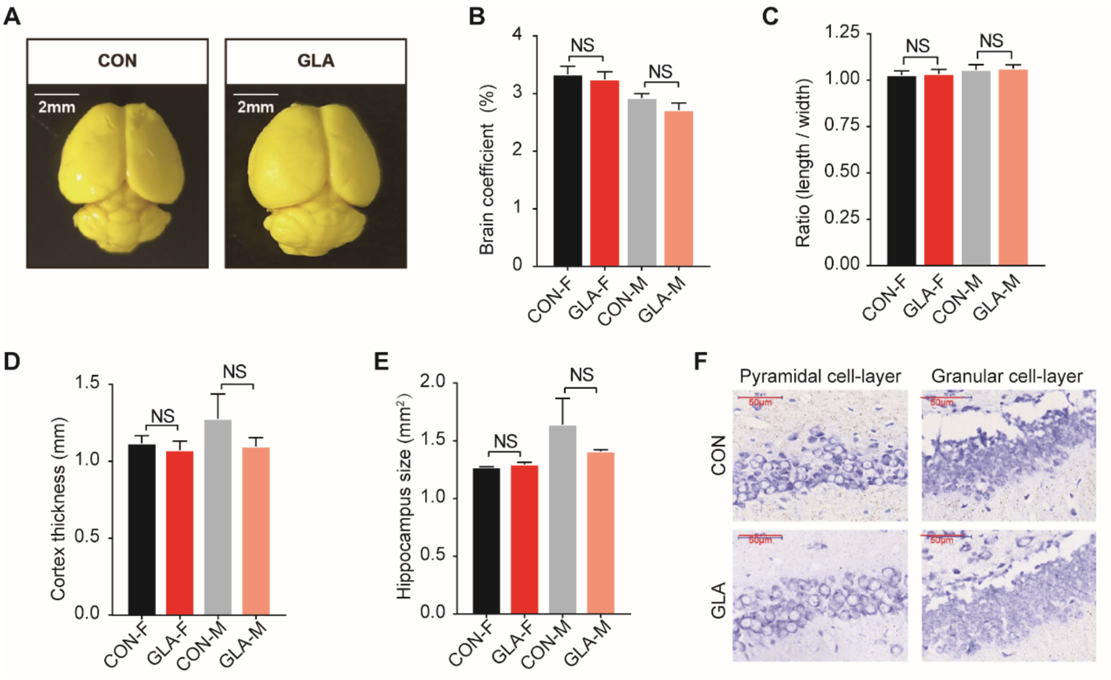

Figure S3. Brain morphology. (A) Representative whole brain images from CON and GLA groups. Scale bar, 2 mm. (B) Brain coefficient. (C) Brain length to width ratio. (D) Prefrontal cortical thickness. (E) Hippocampal size. (F) Representative Nissl stained images of pyramidal cell layer and granular cell layer in CON and GLA groups. Scale bar, 50 μm. For quantitative panels, CON-F and CON-M indicate offspring from control groups, and GLA-F and GLA-M indicate offspring from GLA exposed groups. Data are mean ± SEM. NS, not significant.

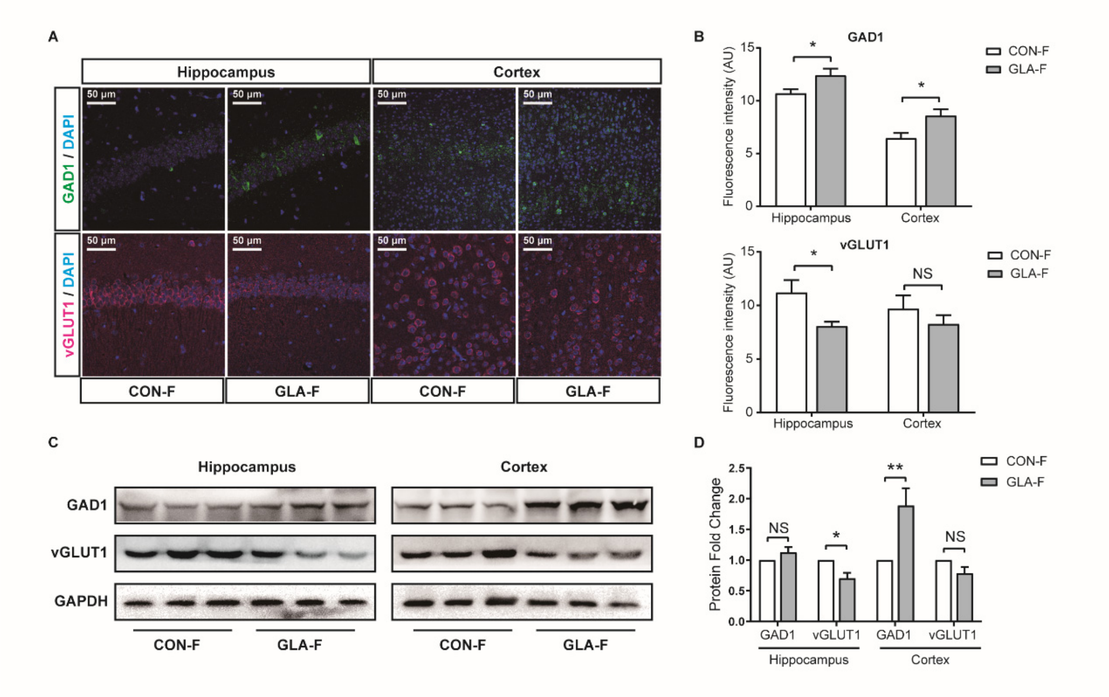

Figure S4. GAD1 and vGLUT1. (A) Representative immunofluorescence images of GAD1 (green) and vGLUT1 (magenta) with DAPI counterstain (blue) in hippocampus and prefrontal cortex from CON-F and GLA-F offspring. Scale bar, 50 μm. (B) Quantification of fluorescence intensity for GAD1 and vGLUT1. (C) Representative immunoblots of GAD1 and vGLUT1 in hippocampus and prefrontal cortex, with GAPDH as a loading control. (D) Quantification of immunoblot band intensity shown as protein fold change relative to CON-F. Bars represent mean ± SEM. NS, not significant; * $P < 0.05$; ** $P < 0.01$.

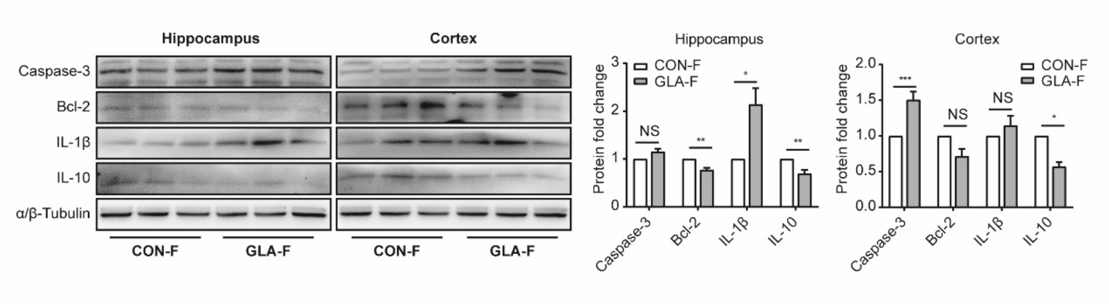

Figure S5. Cytokines and apoptosis. Representative immunoblots and quantification of Caspase-3, Bcl-2, IL-1β, and IL-10 in hippocampus and prefrontal cortex from CON-F and GLA-F offspring at 5 weeks. α/β Tubulin was used as a loading control. Quantification is shown as protein fold change relative to CON-F. Bars represent mean ± SEM. NS, not significant; * $P < 0.05$; ** $P < 0.01$; *** $P < 0.001$.
